# Catastrophic health expenditure among single empty-nest elderly with multimorbidity in rural Shandong, China: the effect of co-occurrence of frailty

**DOI:** 10.1186/s12939-020-01362-6

**Published:** 2021-01-07

**Authors:** Zhengyue Jing, Jie Li, Pei Pei Fu, Yi Wang, Yemin Yuan, Dan Zhao, Wenting Hao, Caiting Yu, Chengchao Zhou

**Affiliations:** 1 Centre for Health Management and Policy Research, School of Public Health, Cheeloo College of Medicine, Shandong University, Jinan, 250012 China; 2 NHC Key Lab of Health Economics and Policy Research, Shandong University, Jinan, 250012 China

**Keywords:** Physical multimorbidity, Frailty, Catastrophic health expenditure, Living alone, China

## Abstract

**Background:**

Previous studies have indicated that older adults with multimorbidity had higher risk of incurring catastrophic health expenditure (CHE). However, the effect of co-occurrence of frailty on CHE among single empty-nest elderly with multimorbidity remains unclear. This study aims to explore the effect of co-occurrence of frailty on CHE among single empty-nest elderly with multimorbidity, and whether this effect is moderated by economic status.

**Methods:**

A cross-sectional household survey of the older adults in 2019 in Shandong province, China. A total of 606 single empty-nest elderly aged 60 years or older were included in this study. CHE was defined as the out-of-pocket payments for health care that equals or exceeds 40% of the household’ s capacity to pay. Logistic regression models are employed to examine the effect of co-occurrence of frailty on CHE among single empty-nest elderly with multimorbidity. The interaction term is introduced to explore the economic status difference in this effect.

**Results:**

The CHE incidence for single empty-nest elderly with multimorbidity alone is 64.2%, and the co-occurrence of frailty results in an increase by almost 1.3 times (84.0%) in CHE incidence among single empty-nest elderly with multimorbidity. The co-occurrence of frailty increases the risk of incurring CHE among the single empty-nest elderly with multimorbidity, with the odds of incurring CHE increased by 3.19 times (OR = 3.19; *P* = 0.005). Furthermore, the interaction analysis shows that the effect of co-occurrence of frailty on CHE among single empty-nest elderly with multimorbidity still exist in lower economic status groups (OR = 4.64; *P* = 0.027), but not in higher economic status (OR = 2.76; *P* = 0.062).

**Conclusions:**

This study demonstrates that there is a positive effect of co-occurrence of frailty on the CHE among the single empty-nest elderly with multimorbidity, and this effect varies by economic status. The health policy-makers should reorganize the healthcare system to make it pro-poor, so as to meet the multiple medical demand and reduce the potential economic burden and inequalities of older adults.

**Supplementary Information:**

The online version contains supplementary material available at 10.1186/s12939-020-01362-6.

## Background

China’ s population is aging dramatically. The proportion of the population aged 65 years and over will increase from 11.5% (164million) in 2018 to 16.9% (246 million) in 2030 in China [1]. Simultaneously, with the rapid economic development and urbanization, great changes have taken place in the family structure in China. Following this trend, empty-nest older households have become the main model, accounting for about 50% of the total older households. The number of single empty-nest elderly is also on the rise [2], with an estimated percentage of 10% of the total empty-nest older adult population [3]. This percentage is projected to increase rapidly in the next decades. The single rural empty-nest elderly are more disadvantaged due to the fact that they have lower socio-economic status and limited access to health service than their urban counterparts [4], which has aroused widespread concerns in the whole society.

Population aging is known to be accompanied by the increasing prevalence of chronic diseases. It has been demonstrated that more and more older people are affected by multimorbidity, which refers to the coexistence of two and more chronic conditions in a single individual [5, 6]. A review showed that the prevalence of multimorbidity in older adults (60+) ranged from 6.4 to 76.5% in China [7]. Multimorbidity can not only negatively affect the quality of life of the older adult [8], but also bring high economic burden to the older adult themselves, and also their households. Some previous studies have found that there was a significant positive relationship between multimorbidity and healthcare use and total healthcare cost [9, 10], as well as out-of-pocket (OOP) healthcare spending [11]. Higher OOP healthcare spending may result in financial catastrophe. According to World Health Organization (WHO), when OOP payments for health care that equals or exceeds 40% of the household’ s capacity to pay, the households may face catastrophic health expenditure (CHE). An epidemiological study has demonstrated that older adults with multimorbidity experienced a higher probability of incurring CHE than those without multimorbidity [12].

Nevertheless, multimorbidity can only explain small fraction of high healthcare expenditure in older adults, previous studies have indicated that frailty was associated with the increase of healthcare cost and OOP medical expenditure in older adults after adjusted for multimorbidity [13]. Frailty, as a common geriatric syndrome, is defined as the state of increased vulnerability of health adverse outcomes due to the aging-related decline in reserve and function across multiple-physiological systems [14]. A review reported the prevalence of frailty among community older adults (65+) varies from 5.9 to 17.4% [15].

Although multimorbidity or frailty was known as the independent predicator of high healthcare cost, these two conditions may exist bidirectional relationship or co-occurrence. Indeed, a review demonstrated that that multimorbidity increase the likelihood of reporting frailty and vice versa [16]. Also, the coexistence of physical multimorbidity and frailty was found to be more likely to increase the risk of physical limitations, mortality and other adverse health outcomes in older adults than multimorbidity or frailty status singly [17, 18]. However, the effect of co-occurrence of frailty and multimorbidity on CHE among single empty-nest elderly remains unclear. Furthermore, there is economic status inequality in distribution of CHE incidence and people with poor economic status were more likely to incur CHE than those with higher economic status [12]. Conversely, a study found that the effect of multimorbidity on CHE in lower and higher economic groups is similar [19], while whether the effect of co-occurrence of frailty on CHE among single empty-nest elderly with multimorbidity varies by economic status is also unclear.

Older adults living alone had highest risk of incurring CHE than older adults living with spouse or non-empty-nest older households [20]. Therefore, this study mainly aims to explore the effect of co-occurrence of frailty on CHE among single empty-nest elderly with multimorbidity in rural China, and whether this effect is moderated by economic status. We hypothesize that the effect of co-occurrence of frailty on CHE among single empty-nest elderly with multimorbidity is statistically significant. We further hypothesize that the effect of co-occurrence of frailty on CHE among single empty-nest elderly with multimorbidity varies by economic status.

## Methods

### Data source

This study conducted a household survey of the older adults in 2019 in Shandong province, China. Shandong is located in the east of China, which ranks the second in population size in China, with a number of 107 million people in 2018. The older adults aged 60 years and above accounted for 22.29% of the total population in Shandong in 2018. A multi-stage stratified random sampling method was adopted to select participants, which is explained in detail elsewhere [21]. In this study, 618 respondents who lived alone were selected. Of whom, 12 respondents with missing main variables were excluded, and a total of 606 older adults living alone with completed data were included in the analysis.

### Measurements

#### Outcome variables--catastrophic health expenditure

For those older single households, CHE is defined as when the OOP medical expenditure equals or exceeds 40% of household’ capacity to pay. In this study, the capacity to pay refers to non-food household consumption spending. CHE is assessed by the indicator of incidence and intensity, of which, intensity is calculated by mean gap (MG) and mean positive gap (MPG). The incidence describes the proportion of household facing CHE in all sample households. The MG evaluates the severity of CHE in all sample households, which is defined as the average amount by which payment surpassed the threshold; the MPG evaluates the severity of CHE in households with CHE, which is defined as the payments in excess of the threshold in household that exceed the threshold. The calculation methodology of CHE was introduced in detail by Xu K et al. [22] and Wagstaff et al. [23] elsewhere.

#### Physical multimorbidity

In this study, chronic health condition was defined by the World Health Organization (WHO) as being of long duration (usually last 1 year or longer), generally slow progression and not passed from person to person. Physical chronic conditions of the respondents were measured by the following self-reported questions: “Have you ever been diagnosed with a chronic disease by a physician?”. If the answer was ‘yes’, the respondents would be further asked the questions that “How many chronic diseases have you ever been diagnosed by a physician?” In order to ensure the accuracy of the interview information, the trained interviewers with medical knowledge will further asking the help of village doctors to confirm the chronic conditions information in the chronic disease case management system in the sampling villages. The physical multimorbidity in this study refers to one individual with two or more chronic conditions.

#### Frailty

The measurement of frailty was based on the frailty phenotype proposed by Fried and colleagues [24], which include the weight loss, exhaustion, weakness, low physical activity and slowness. Of which, the weights loess was measured by the unintentional of 5% or more of weights in the last year; exhaustion was measured by the two questions that included in the Center for Epidemiologic Studies-Depression scale, and the corresponding answer was most of time was defined as exhaustion; weakness was measured by handgrip strength that using handheld dynamometer, adjusted for gender and body mass index; low physical activity was assessed by using the international Physical Activity Questionnaire (short version) [25]; slowness was measured by the time of 15 ft. walking, adjusted for height and sex. The physical frailty in this study was classified into frailty (defined as positive for three or more frailty criteria) and without frailty.

Furthermore, based on the number of chronic conditions and frailty status, the health states of the respondent were divided into four categories: (1) no chronic conditions; (2) one chronic condition; (3) physical multimorbidity and without frailty syndrome; (4) physical multimorbidity and co-occurrence of frailty syndrome.

#### Covariate variables

We dichotomized annual household expenditure at a cut-point of 6430yuan RMB (median of expenditure/50th percentile), and finally household economic status was divided into two categories: low economic status (< 6430 yuan RMB) and high economic status (> 6430 yuan RMB). Inpatient service utilization was assessed using the question: “Have you ever had used inpatient service in the past 12 months?”, and the response included “yes” or “no”. Other covariate variables also include sex, age, education, alcohol drinking, cigarette smoking, and physical exercise.

#### Statistical analysis

The SPSS version 22.0 was used to analyze the data. Descriptive analysis was employed to describe the basic characteristics of the respondents using the indicator of mean (standard deviation) or frequencies (percentage). Chi-square tests were used to compare the differences between categorical variables, ANOVA analysis was used to examine the differences for continuous variables. The sensitivity analysis by using different thresholds (20, 30, 40, 50, 60%) was also used in descriptive analyses. Univariate and multivariate logistic regression models were employed to explore the effect of co-occurrence of frailty on CHE among single empty-nest elderly with multimorbidity, which tested the first hypothesis. Moreover, in order to test the second hypothesis (the economic status difference in this effect), the interactive terms between multimorbidity and frailty and economic status was introduced into logistic regression model and if the interactive term was statistically significant, we conducted a stratified analysis of this effect by low and high economic status.

## Results

### Basic characteristic of respondents

Table 1 shows the basic characteristic of the respondents. A total of 606 respondents included in this study, with a mean age of 73.1 years. Majority of them are female (67.3%), illiterate (51.7%), do not smoke (72.1%) or drink alcohol (73.4%), take physical exercise (60.7%), do not use inpatient service (77.4%). The Supplementary Table 1 shows the details of the components of household expenditure. The mean OOP payments for health care is 3660yuan RMB (with a median of 1000yuan RMB), which accounts for 57.1% of the average household capacity to pay.

**Table 1:** Demographic characteristics of the single empty-nest elderly (60+) in rural Shandong, China, 2019

Characteristics	Total	No Chronic Condition	One Chronic Conditions	Multimorbidity	Multimorbidity + frailty	***P***-value
**Total**	606 (100.0)	137 (22.6)	240 (39.6)	173 (28.6)	56 (9.2)	
**Sex**						0.070
Male	198 (32.7)	50 (36.5)	88 (36.7)	46 (26.6)	14 (25.0)	
Female	408 (67.3)	87 (63.5)	152 (63.3)	127 (73.4)	42 (75.0)	
**Age (Years)**	73.1 ± 6.5	73.3 ± 6.6	73.4 ± 6.6	72.4 ± 6.0	72.9 ± 6.5	0.036
**Educational attainment**						0.548
Illiterate	313 (51.7)	78 (56.9)	122 (50.8)	86 (49.7)	27 (48.2)	
Primary school	217 (35.8)	59 (43.1)	118 (49.2)	87 (50.3)	29 (51.8)	
Middle school or above	76 (12.5)					
**Household economic status**^a^						0.005
Low(Q1/Q2)	303 (50.0)	83 (60.6)	123 (51.2)	70 (40.5)	27 (48.2)	
High(Q3/Q4)	303 (50.0)	54 (39.4)	117 (48.8)	103 (59.5)	29 (51.8)	
**Cigarette smoking**						0.225
Never smoker	437 (72.1)	98 (71.5)	171 (71.2)	124 (71.7)	44 (78.6)	
Current smoker	125 (20.6)	34 (24.8)	52 (21.7)	31 (17.9)	8 (14.3)	
Former smoker	44 (7.3)	5 (3.7)	17 (7.1)	18 (10.4)	4 (7.1)	
**Alcohol drinking**						0.013
No	469 (77.4)	97 (70.8)	179 (74.6)	145 (83.8)	48 (85.7)	
Yes	137 (22.6)	40 (29.2)	61 (25.4)	28 (16.2)	8 (14.3)	
**Physical exercise**						0.020
No	238 (39.3)	49 (35.8)	97 (40.4)	60 (34.7)	32 (57.1)	
Yes	368 (60.7)	88 (64.2)	143 (59.6)	113 (65.3)	24 (42.9)	
**Inpatient service**						0.010
No	469 (77.4)	117 (85.4)	190 (79.2)	122 (70.5)	40 (71.4)	
Yes	137 (22.6)	20 (14.6)	50 (20.8)	51 (29.5)	16 (28.6)	

^a^Using the quartile method, Quartile 1 (Q1) is the poorest and quartile 4 (Q4) is the richest

### Incidence and intensity of CHE

Table 2 shows the incidence and intensity of CHE by number of chronic conditions and frailty status. At the 40% threshold, the overall CHE incidence is 49.8%, the overall MG and MPG of the respondents is 13.5 and 27.2%, respectively. The CHE incidence is increased with the number of chronic conditions, the CHE incidence is 64.2% for respondents with only multimorbidity at the 40% threshold. The MG and MPG shows a rising trend with the number of chronic conditions, of which, the MP and MPG (using 40% threshold) is 17.2 and 26.9% for those with only multimorbidity, respectively.

**Table 2:** Incidence and intensity of catastrophic health expenditure among the single empty-nest elderly in rural Shandong, China,2019

Catastrophic health expenditure	Thresholds				
	20%	30%	40%	50%	60%
**Head count (%)**					
Total	67.8	59.4	49.8	38.4	28.9
None chronic condition	35.0	29.2	24.8	19.7	16.8
One chronic condition	68.8	57.9	45.8	33.3	25.0
Multimorbidity	84.4	76.3	64.2	51.5	38.7
Multimorbidity+ frailty	92.9	87.5	84.0	64.3	44.6
*P*-value	< 0.001	< 0.001	< 0.001	< 0.001	< 0.001
**Mean catastrophic payment gap (%)**					
Total	25.4	19.1	13.5	9.1	5.7
None chronic condition	13.4	10.2	7.5	5.2	3.3
One chronic condition	23.9	17.6	12.3	8.2	5.3
Multimorbidity	32.4	24.4	17.2	11.4	6.8
Multimorbidity+ frailty	39.5	30.5	21.9	17.2	9.1
**Mean positive gap (%)**					
Total	37.4	32.1	27.2	23.6	19.6
None chronic condition	38.2	25.1	30.2	26.4	19.9
One chronic condition	34.8	30.4	26.9	24.7	21.3
Multimorbidity	38.3	32.0	26.9	21.3	17.6
Multimorbidity+ frailty	42.5	34.9	26.1	22.2	20.5

Moreover, the co-occurrence of frailty results in highest CHE incidence among single empty-nest elderly with multimorbidity at all of the different thresholds. At the 40% threshold, the CHE incidence for respondents is 84.0% when co-occurring frailty, which is 1.3 times higher than those with multimorbidity only.

### The effect of co-occurrence of frailty on the CHE among single empty-nest elderly with multimorbidity

Table 3 presents the effect of co-occurrence of frailty on the CHE among single empty-nest elderly with multimorbidity. In model 2, after controlling for sex, age, education, economic status, alcohol drinking, cigarette smoking, physical exercise, and inpatient service, the number of chronic conditions is significantly associated with CHE, and the effect of co-occurrence of frailty on the CHE among the single empty-nest elderly both with multimorbidity is statistically significant. Specifically, compared with older adults living alone with multimorbidity only, those without chronic condition (OR = 0.21; *P* < 0.001) and with one chronic condition (OR = 0.51; *P* = 0.003) are less likely to suffer from CHE. Moreover, the co-occurrence of frailty increases the risk of incurring CHE among the single empty-nest elderly with multimorbidity, with the odds of incurring CHE increased by 3.19 times (OR = 3.19; *P* = 0.005).

**Table 3:** The effect of co-occurrence of frailty on the catastrophic health expenditure among single empty-nest elderly with multimorbidity in rural Shandong, China,2019

	Model 1 (No covariates)			Model 2 (Covariates)		
Characteristic	OR	***P***-value	95%CI	OR	***P***-value	95%CI
**Health states**						
None chronic condition	0.18	< 0.001	0.11–0.30	0.21	< 0.001	0.12–0.36
One chronic condition	0.47	< 0.001	0.32–0.71	0.51	0.003	0.33–0.79
Multimorbidity (ref)	1.0			1.0		
Multimorbidity+ frailty	2.92	0.007	1.34–6.35	3.19	0.005	1.41–7.24
**Sex**						
Male				1.0		
Female				1.23	0.404	0.75–2.02
**Age (Years)**				1.05	0.003	1.01–1.08
**Educational attainment**						
Illiterate				1.0		
Primary school				1.01	0.957	0.67–1.52
Middle school or above				0.71	0.290	0.38–1.34
**Household economic status**^a^						
Low(Q1/Q2)				1.0		
High(Q3/Q4)				1.20	0.353	0.82–1.77
**Cigarette smoking**						
Never smoker				1.0		
Current smoker				0.64	0.117	0.37–1.15
Former smoker				2.56	0.020	1.16–5.65
**Alcohol drinking**						
No				1.0		
Yes				0.52	0.017	0.31–0.89
**Physical exercise**						
No				1.0		
Yes				1.05	0.808	0.72–1.53
**Inpatient service**						
No				1.0		
Yes				2.98	< 0.001	1.87–4.75

^a^Using the quartile method, Quartile 1 (Q1) is the poorest and quartile 4 (Q4) is the richest

### The economic status difference in the effect of co-occurrence of frailty on CHE among single empty-nest elderly with multimorbidity

Table 4 presents that the effect of co-occurrence of frailty on CHE among single empty-nest elderly with multimorbidity varies by economic status (*P* < 0.05). The stratified analyses further show that in low household economic status, the effect of co-occurrence of frailty on the CHE among the single empty-nest elderly with multimorbidity still existing (OR = 4.64; *P* = 0.027). However, in high economic status, the co-occurrence of frailty has no effect on the CHE among the single empty-nest elderly with multimorbidity (OR = 2.76; *P* = 0.062).

**Table 4:** The economic status difference in the effect of co-occurrence of frailty on catastrophic health expenditure among single empty-nest elderly with multimorbidity in rural Shandong, China, 2019

Characteristic	Model 1			Model 2			Model 3		
	Interaction^a^			Low economic status			High economic status		
	OR	***P***-value	95%CI	OR	***P***-value	95%CI	OR	***P***-value	95%CI
**Health states**									
None chronic condition	0.12	< 0.001	0.06–0.27	0.11	< 0.001	0.05–0.24	0.39	0.020	0.05–0.24
One chronic condition	0.42	0.010	0.22–0.81	0.38	0.005	0.20–0.75	0.61	0.100	0.33–1.10
Multimorbidity (ref)	1.0			1.0			1.0		
Multimorbidity+ frailty	3.87	0.047	1.02–14.71	4.64	0.027	1.19–18.2	2.76	0.062	0.95–8.00
**Sex**									
Male	1.0			1.0			1.0		
Female	1.34	0.249	0.81–2.2	0.96	0.920	0.47–1.97	1.54	0.221	0.77–3.32
**Age (Years)**	1.04	0.003	1.02–1.08	1.08	0.001	1.04–1.13	1.01	0.481	0.97–1.06
**Educational attainment**									
Illiterate	1.0			1.0			1.0		
Primary school	1.01	0.946	0.67–1.52	1.05	0.864	0.58–1.90	0.99	0.985	0.55–1.79
Middle school or above	0.65	0.192	0.34–1.24	0.48	0.233	0.13–1.63	0.78	0.534	0.36–1.69
**Cigarette smoking**									
Never smoker	1.0			1.0			1.0		
Current smoker	0.65	0.121	0.37–1.12	0.74	0.486	0.31–1.73	0.56	0.133	0.27–1.19
Former smoker	2.21	0.070	0.94–5.22	2.79	0.065	0.94–8.31	2.66	0.112	0.79–8.89
**Alcohol drinking**									
No	1.0			1.0			1.0		
Yes	2.60	0.113	0.79–8.52	0.48	0.052	0.23–1.01	0.57	0.154	0.26–1.24
**Physical exercise**									
No	1.0			1.0			1.0		
Yes	1.00	0.990	0.68–1.47	1.35	0.277	0.78–2.33	0.79	0.426	0.46–1.39
**Inpatient service**									
No	1.0			1.0			1.0		
Yes	2.89	< 0.001	1.81–4.59	1.63	0.189	0.79–3.37	4.11	< 0.001	2.21–7.66
**Household economic status**^b^									
Low(Q1/Q2)	1.0								
High(Q3/Q4)	0.87	0.684	0.44–1.72						
**Health states× Household economic status**									
None chronic condition× High	3.12	0.040	1.05–9.26						
One chronic condition× High	1.39	0.457	0.58–3.34						
Multimorbidity× High	1.0								
(Multimorbidity +frailty) × High	0.70	0.684	0.13–3.84						

^a^The interaction between health states and household economic status

^b^Using the quartile method, Quartile 1 (Q1) is the poorest and quartile 4 (Q4) is the richest

## Discussion

In this study, we find that 49.8% of the single empty-nest elderly in rural China suffered from CHE at the threshold of 40%. It is much higher than the reported 17.0% among the older people (60+) households in a study based on the data from the China Health and Retirement Longitudinal Study of 2012 [26]. It is higher than 14.4% in the general rural households using a nation-wide data [27], and 12.1% in the general rural households in the same province as the current study in 2013 [28]. Also, other studies reported that the MG and MPG were 6.21 and 24.19% in older adult households [12], and 3.7 and 22.2% in the general rural households [29].,respectively. Compared with those studies using the same threshold, the incidence and intensity in the current study is higher than those among general rural households or general older adult households. The possible explanation is that we only focus on rural single empty-nest elderly. The empty-nest elderly had lower economic status than the non-empty-nest elderly, especially for the single rural empty-nest elderly, they had lower income and receive less economic support from their children [30], so they were more vulnerable to health risk. Meanwhile, previous studies have indicated that empty-nest elderly had poorer heath status (higher prevalence of chronic diseases) and higher health needs than the non-empty-nest elderly [31, 32], which might incur higher health service use and medical expenditure, and eventually increased the likelihood of suffering from CHE. This study finds that the number of chronic conditions is significantly associated with CHE in older people living alone, which is consistent with previous study [19]. A possible explanation for this relationship might be that the higher OOP expenditure caused by multiple chronic conditions pushes older adults into heavy economic burden and CHE. A previous study in China demonstrated a positive relationship between the number of chronic conditions and outpatient OOP spending [11]. Many studies conducted in other countries also showed the same results [33–35], one of which further revealed that each additional chronic condition was correlated with the risk of falling into financial catastrophe increased by 46% in older adults [33].

In line with our hypothesis, we further find that there is a positive effect of co-occurrence of frailty on the CHE among the single empty-nest elderly with multimorbidity, with the odds of incurring CHE increased by 3.19 times. A plausible explanation for this finding might be that the existence of frailty stimulates the higher healthcare expenditure and OOP expenditure for single empty-nest elderly with multimorbidity, which tends to push them into a heavier economic burden and CHE. Although there may be co-existence or interaction between multimorbidity and frailty, frailty was found to be an independent risk factor for increasing healthcare expenditure above and beyond that explained by multimorbidity. For instance, a study indicated that frailty was associated with increased medical spending after controlling for multimorbidity among Chinese older adults [36]. In some countries, such as the United states, Germany, Korea, several studies also demonstrated that the increasing healthcare expenditure as well as OOP medical cost increased with the level of frailty in older adults after accounting for multimorbidity [13, 37, 38]. Based on this fact, the co-occurrence of frailty may cause higher OOP medical expenditure and eventually increase the risk of CHE for single empty-nest elderly with only multimorbidity. In addition, a study found that older adults with both multimorbidity and frailty were more likely to use more than three drugs than those with only multimorbidity [17], hence the other interpretation is that the single empty-nest elderly with both multimorbidity and frailty had higher probability of facing adverse health outcomes and paying higher medical cost to maintain health status than those with only multimorbidity, which may ultimately impose higher financial burden and incur CHE.

Furthermore, this study finds that the single empty-nest elderly in lower economic status with both multimorbidity and frailty were more likely to experience CHE than those with multimorbidity alone, whereas this association in higher economic status is not statistically significant. Zhao et al. found that among the adults covered by Urban Resident Basic Medical Insurance (URBMI), the effect of multimorbidity on CHE was increased in lower economic groups [19]. Different from those in urban areas, the rural older adults cannot receive a retirement pension and have limited financial source. In addition to the maintenance from the children, labor activity is the main source of income for the rural older adults living alone [39]. A 4 years of follow-up study found that frailty co-occurring with other syndromes (multimorbidity or dependency) was the strongest predicator of increasing the risk of physical limitations [17]. Thus, we speculated that the co-occurrence of frailty may result in a decline/loss of the ability of labor activity among the rural single empty-nest elderly with multimorbidity and then an inability to earn income. The rural single empty-nest elderly in low economic status were more likely to be vulnerable to such health shocks than those in higher economic status [40], and eventually suffer from CHE due to the limited income combined with high healthcare expenditure caused by multimorbidity and frailty.

The findings in the current study imply that health policy-makers should reorganize the healthcare system, enhance coordination across different health care system levels, develop and implement comprehensive and long-term care plans and clinical guidelines for people with multiple conditions, focusing on the management of the coexisting chronic conditions and frailty in the elderly, so as to meet the diverse medical demand of the older adults. Next, the primary health care providers in rural China should not only strengthen the management of multiple chronic cases, but also to prevent and delay the occurrence of frailty. It is imperative to develop frailty screening measures (for example, incorporating the frailty assessment into the current free physical examination programs for the older adults), and encourage the exploration of risk factors for frailty in the older adults, and finally formulate frailty interventions measures. Also, the government should provide financial protection measures for the rural single empty-nest elderly to reduce the potential economic burden and inequalities caused by multiple chronic diseases and frailty, especially among the low-income groups. For example, increase subsidies for low-income rural single empty-nest elderly; develop health insurance scheme and medical assistance programs, to heighten the ceiling line and expand to cover more kinds of chronic diseases and physical frailty.

This study has some limitations. First, the healthcare spending and household income are self-reported, which may lead to recall bias, especially in older adult population. Second, the number of chronic conditions also are self-reported, despite the investigator with medical knowledge verified the chronic conditions with village doctors, there may still exist bias. Third, the data are cross-sectional and could not draw the causal relationship, which would be remedied in the follow-up study.

## Conclusions

This study demonstrates that the co-occurrence of frailty results in an increase by almost 1.3 times in CHE incidence among single empty-nest elderly with multimorbidity. Moreover, the co-occurrence of frailty increases the risk of incurring CHE among single empty-nest elderly with multimorbidity, and this effect still exists in lower economic status groups, but not in higher economic status groups. Policy makers should develop medical assistance programs targeting the rural single empty-nest elderly to reduce the potential economic burden and inequalities caused by multiple chronic diseases and frailty, particularly protect those in lower economic status from higher risk of CHE.

## Supplementary Information

**Additional file 1: Supplementary Table 1**. Distribution of capacity to pay and OOP costs for health care among the single empty-nest elderly (60+) in rural Shandong, China, 2019

## Data Availability

All the research data is available from the correspondence author upon reasonable request.

## Abbreviations

CHE: Catastrophic health expenditure
OOP: Out-of-pocket
WHO: World Health Organization
MG: Mean gap
MPG: Mean positive gap
URBMI: Urban Resident Basic Medical Insurance
