# Supplementary material for: Catastrophic health expenditure among single empty-nest elderly with multimorbidity in rural Shandong, China: the effect of co-occurrence of frailty
Source: Int J Equity Health. 2021 Jan 7;20:23. doi: 10.1186/s12939-020-01362-6 (PMC7792165; doi:10.1186/s12939-020-01362-6)
Supplement: Supplementary file 1 — Additional file 1: Supplementary Table 1. Distribution of capacity to pay and OOP costs for health care among the single empty-nest elderly (60+) in rural Shandong, China, 2019 [file 12939_2020_1362_MOESM1_ESM.docx]

**Supplementary Table1** Distribution of capacity to pay and OOP costs for health care among the single empty-nest elderly (60+) in rural Shandong, China,2019

| **Indicators** | **Total** |
| --- | --- |
| Survey frequency | 606 |
| Average OOP costs for health care (yuan RMB) |  |
| Mean (SD) | 3,660(14,538) |
| Median (P_25_; P_75_) | 1,000(300;3000) |
| Average annual household expenditure (yuan RMB) |  |
| Mean (SD) | 9,278(15,970) |
| Median (P_25_; P_75_) | 6,245(3,850;9,930) |
| Average annual household food expenditure (yuan RMB) |  |
| Mean (SD) | 2,866(3,402) |
| Median (P_25_; P_75_) | 2,400(1,200; 3,600) |
| Average household capacity to pay (yuan RMB) |  |
| Mean (SD) | 6,412(15,404) |
| Median (P_25_; P_75_) | 3,560(1,820,6,450) |
| Average OOP payments share of capacity to pay (%) | 57.1 |
| Households with catastrophic health expenditure (%) | 49.8 |
